# Observation of Bridging Chlorine in Vitreous SiO_2_

**DOI:** 10.1021/acs.jpcb.6c02141

**Published:** 2026-09-29

**Authors:** Lindsay M. Harrison, Adam R. Sarafian, Randall E. Youngman, Craig D. Nie, Matthew E. McKenzie, Alisha N. Clark

**Affiliations:** † Department of Earth Science, University of Colorado, Boulder, Boulder, Colorado 80309, United States; ‡ 2690 Corning Incorporated, Science & Technology, Corning, New York 14831, United States

## Abstract

The short-range to mid-range structure of inorganic glasses profoundly impacts their physical properties. In this work, we have used multiple spectroscopic methods to examine the change in the network structure of amorphous silica with addition of Cl. Unlike analogous F-doped silicas, where the bonding of halogen leads to terminal Si–F groups, NMR and Raman data for Cl-doped v-SiO_2_ indicate bridging between silica tetrahedra, preserving rather than reducing network connectivity and contributing to their elastic stiffness and viscosity behaviors. These bridging Cl atoms make v-SiO_2_ stiffer while having the same effect on density behavior per mol % dopant as F in v-SiO_2_ and thus may be a desirable attribute in tailoring v-SiO_2_ for optical applications.

## Graphical abstract

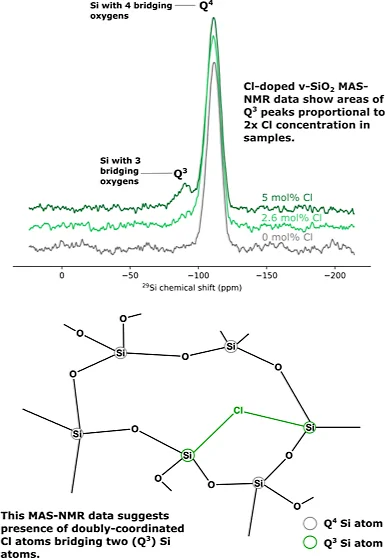

## Introduction

Vitreous silica (v-SiO_2_) is an amorphous solid comprising a network of interconnected SiO_2_ tetrahedra joined by bridging oxygens. Connectivity of the network is related to the distribution of 3- to 7-member rings, which are composed of silicate tetrahedra (SiO_4/2_, where 4 represents all O atoms bonded to a Si atom, and 2 represents how many Si atoms share each O) that bond together via a shared, bridging oxygen (BO).^1^ The addition of dopants (e.g., F, OH) affects the distribution of ring sizes by the formation of terminal (nonbridging) bonds that either displace or terminate a BO.^2,3^ Depolymerization in amorphous silicates results in decreased viscosity, increased structural relaxation, and elastic softening.^4–6^ These glass properties are critical when considering the fabrication and performance of v-SiO_2_ and doped silica in optical applications, especially in optical fiber.

Halogens, including F and Cl in particular, are used in the silica laydown process: F as a dopant for optical fiber and Cl as a drying agent to remove impurities.^7^ It is generally understood that the addition of F depolymerizes the silicate network by the displacement of a BO on a 4-coordinated, or Q^4^, Si atom, typically forming a SiO_3/2_F unit.^3,4,8^ Hypervalent silicon atoms with excess electrons in the valence shell, such as SiO_4/2_F and SiO_3_F_2_, have been predicted by molecular dynamics (MD) simulations and observed in nuclear magnetic resonance (NMR) spectroscopy.^3,9^ Halogens have been observed to form hypervalent units in organic systems, but the lack of observations in glassy systems has yet to support the hypothesized hypervalent Si atoms.^10^

Chlorine lies below F in the periodic table and has been observed to have similar effects to F in v-SiO_2_; compared to undoped v-SiO_2_, the addition of Cl reduces viscosity, lowers elastic moduli, and facilitates structural relaxation.^4,5,11^ Previous solid-state NMR studies on silicate and aluminosilicate glasses containing ^35^Cl show bonding of Cl solely to network modifier cations as opposed to the network-forming silicon atoms.^12^ In irradiated glass lacking network modifier cations, Cl atoms form “decorated” clusters of Si–Cl terminal bonds that encircle a silica tetrahedron defect.^13^ While these studies have shown the ability of Cl to bond to network modifier atoms, the coordination of Cl to a network former atom, much less the effects of Cl on the network of silicate tetrahedra, especially in unirradiated silica, is not as well studied. There are numerous technological advantages to Cl in v-SiO_2_ such as increasing refractive index, lowering Rayleigh scattering, and suppression of devitrification.^14–16^ Furthermore, because recent research has found that Cl alters the midrange order in favor of fewer large rings and more small rings, it is important to understand how Cl affects the silicate network.^17^

In this paper, we characterize the bonding behavior of Cl atoms in the silicate network by using ^29^Si magic-angle spinning nuclear magnetic resonance (MAS NMR) spectroscopy, Raman spectroscopy, MD simulations, and viscosity measurements. ^29^Si MAS NMR allows us to examine the impact of Cl on Si coordination and bonding; Raman spectroscopy provides complementary information about medium-range order structures. Together, they aid in understanding the structural character of Cl in v-SiO_2_. In conjunction with spectroscopic measurements, MD simulations provide predictions for spectroscopic observations for different bonding configurations of Cl. We find that these results, taken together with recent work on the elastic moduli and density of Cl-doped v-SiO_2_, are consistent with the presence of bridging Cl, that is, a Si–Cl–Si covalent bond where a single Cl atom replaces two BO and results in the bridging of two silica tetrahedra. Furthermore, we present viscosity measurements that suggest that bridging Cl may not only be a glassy phenomenon but may exist in the liquid phase.

## Methods

### Sample Synthesis

A suite of type IV v-SiO_2_ samples made by flame hydrolysis containing chlorine were prepared at Corning Incorporated (Supporting Information, Data S1, S7, and S8). Samples ranged from pure SiO_2_, with lower than detectable OH and <10 ppb metallic impurities, up to 5.6 mol % Cl. These samples are made by the laydown process, in which the SiCl_4_ precursor is vaporized and then combusted using an oxygen–hydrogen flame, producing silica soot that is deposited onto a rotating rod, forming a blank. The resulting blank is then heated with a specific partial pressure of Cl to ensure homogeneous Cl distribution and stretched into ∼1 cm diameter canes. We compare the Cl-doped silicas to a reference type IV fused silica (glass code HPFS 8655) with low impurity content (<1 ppm of OH and <10 ppb metallic impurities). Dopant concentrations were determined using electron probe microanalysis (EPMA). Density was determined via Archimedes’ method using sufficient mass for samples (5 g), which were broken off from canes of samples.

### Electron Probe Microanalysis Measurements

Dopant concentrations in v-SiO_2_ samples by EMPA measurements. Samples were mounted in epoxy, polished in cross-section, and carbon-coated prior to analysis. Electron microprobe analyses were carried out using a JEOL JXA-8500 Hyperprobe field-emission instrument. F was analyzed using a TAP diffracting crystal, whereas Cl was analyzed using a PET diffracting crystal. As is typical in routine measurements, SiO_2_ was calculated by difference. Calibration was performed using single-crystal MgF_2_ and USNM R6600-1 scapolite as standards. Analyses were conducted at 15 keV and 200 nA. Line scans were acquired using a defocused 20 μm spot at 50 μm step intervals.

### Nuclear Magnetic Resonance Spectroscopy

We performed ^29^Si magic-angle spinning nuclear magnetic resonance (MAS NMR) spectroscopy on Cl-containing samples with 2.6 mol % and 5.6 mol % Cl. Even though it is possible to perform NMR spectroscopy on ^35^Cl, the spectrum provides a much lower signal-to-noise ratio than that of ^29^Si in addition to the presence of broad peaks; with low Cl concentrations as in these samples, these issues are compounded.^18^ Because Cl will primarily bond to Si in v-SiO_2_, analysis of the ^29^Si nucleus is a good proxy for the bonding environment of Cl in v-SiO_2_.^13,19^ The abundances of Q^*x*^ species—where *x* represents the number of BOs coordinated to Si—are directly proportional to the integrated peak area of the distribution functions that comprise the MAS NMR spectra.

NMR spectroscopy was performed on crushed Cl-doped silica samples using a VNMRS Agilent spectrometer and a Chemagnetics/Varian T3 5 mm double resonance MAS NMR probe. An Oxford 11.7 T wide-bore magnet gives a ^29^Si resonance frequency of 99.2758 MHz. The sample was loaded into a 5 mm zirconia rotor with Teflon spacers and an end-cap, and the sample was spun at 9.0 kHz using compressed nitrogen. We signal-averaged 130 and 100 scans for the 2.6 mol % and 5.6 mol % Cl v-SiO_2_, respectively. Spectra were obtained using direct polarization MAS NMR with a π/2 pulse width of 4.8 μs and a recycle delay of 6000 s. We processed the spectra using VnmrJ, a commercial software package, and frequency-referenced them to an external tetramethylsilane (TMS) standard at 0.0 ppm.

### Molecular Dynamics Simulations

Modifications to the Pedone, Malavasi, Menziani, Cormack, and Segre (PMMCS) force field were necessary to accurately model the interactions of dopants (F and Cl) in the v-SiO_2_ system.^20^ In summary, to make the force field somewhat commensurate with these elements, we used a charged Lennard-Jones (LJ) potential. Energy versus separation curves were calculated for model clusters of (OH)_3_-Si-X (X = F or Cl) where the (OH)_3_ group approximates three bridging oxygens typical of the silicate network. The LJ parameters were fit to these curves, with sigma (σ) adjusted to match experimental atomic distances and epsilon (ε, the potential well depth) calibrated to ensure compatibility with quantum mechanical results. The final LJ parameters for F and Cl are provided in Table S1. The methods summarized here are explained in further detail in recent work.^17^

### Viscosity Measurements

Viscosities of the type IV fused silica canes containing both Cl and F dopants were measured by the cane elongation method (Figure S1). This method measures the velocity of a cane of measured radius (∼4 mm) under a known load as it is heated in a furnace. Viscosity is calculated using the equation

1 $$\eta =\frac{L\times \Delta t\times m\times g}{3\times \Delta l\times \pi r^{2}}$$

where η is the viscosity (in Pa s), *L* is the length of the hot zone (furnace), $\frac{\Delta l}{\Delta t}$ is the velocity, *m* is the mass below the hot zone, *g* is the acceleration due to gravity, and *r* is the radius of the cane.^21,22^ Glass transition temperatures (*T*_g_) were calculated by extrapolating the logarithmic relationship of viscosities up to 10^12^ Pa s (reported in Supporting Information, Table S2).

## Results and Discussion

^29^Si MAS NMR spectra of Cl-doped v-SiO_2_ are plotted in Figure 1a. The coordination environment is interpreted from the chemical shift, which is the ppm shift from the standard (TMS). Spectra for v-SiO_2_ containing two different concentrations of Cl are composed of two Gaussian peaks centered at approximately −92 and −111 ppm; peak fits to the spectra are shown in Figure 1b, and peak areas are shown in Table 1. The prominent peak in each spectrum is centered around −111 ppm and corresponds to Si atoms in Q^4^ tetrahedra, as in pure v-SiO_2_. The weaker resonance is centered at −94 ppm in the 2.6 mol % Cl sample and −91 ppm in the 5.6 mol % Cl sample and is both more intense and more resolved at the higher Cl-doping level. A peak that is downshifted (less negative) is deshielded compared to one with a lower chemical shift (more negative), indicating that the nucleus has lower electron density compared to those that are more shielded. In a covalently bonded solid, such as v-SiO_2_, Q^4^ Si is shielded because it shares 4 covalent bonds with BOs. While the chemical shift of the Q^4^ peak stays constant for both v-SiO_2_ samples, the Q^3^ peak shifts downfield from −94 to −91 in 2.6 mol % Cl and 5.6 mol % Cl, respectively. Consistent with this interpretation, a Q^3^ Si resonance has been identified with the presence of fluorine in SiO_3/2_F around −94 ppm,^8^ so we posit that the similar ^29^Si NMR resonance in our data indicates the presence of SiO_3/2_Cl groups—that is, a Si tetrahedron with 3 BOs and a Cl atom (Figure 1b inset).

**Figure 1:**
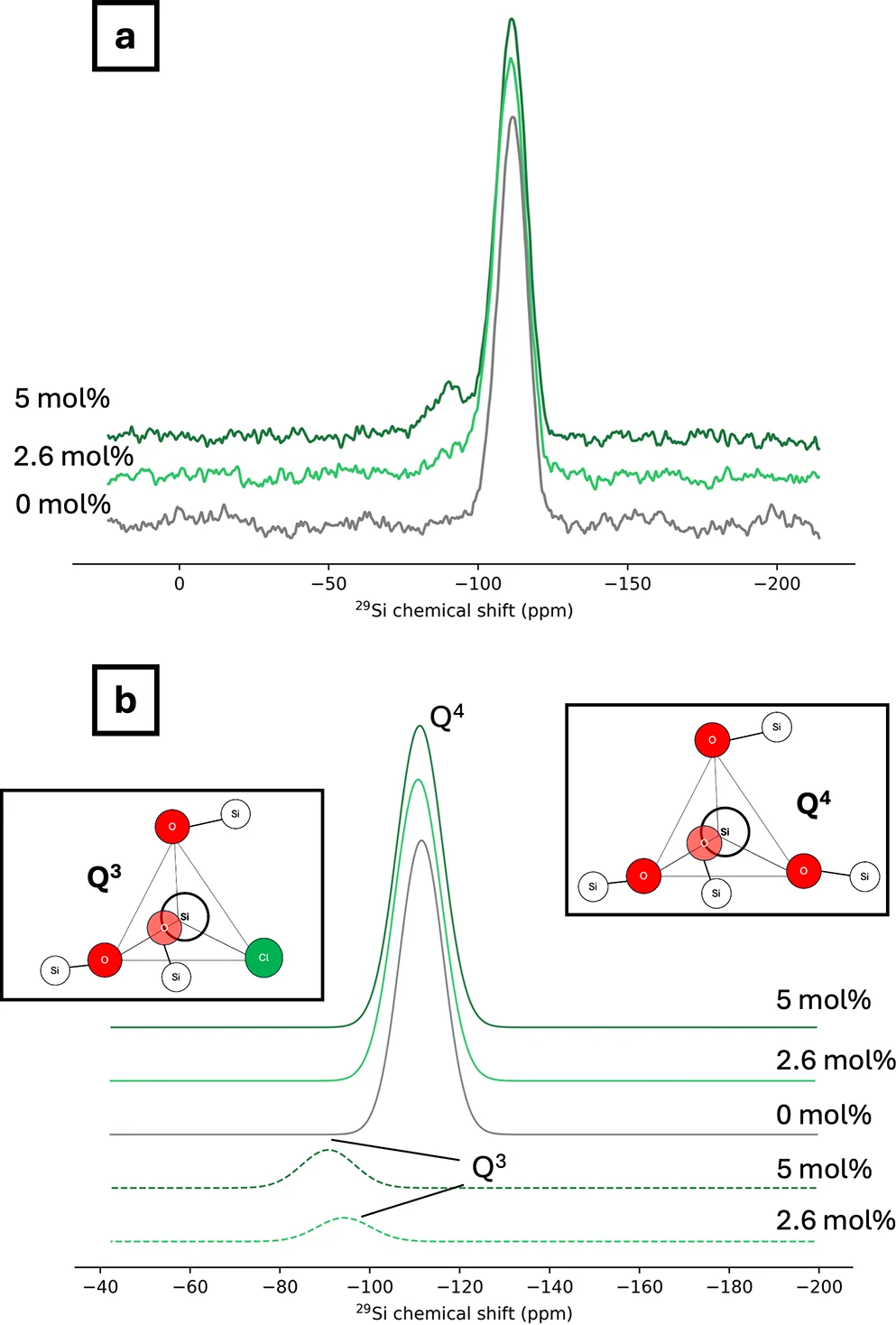
(a) ^29^Si MAS NMR spectrum and (b) fitted peaks at −111 ppm, labeled Q^4^, and at ∼−91 ppm, labeled Q^3^ for 0 mol % (gray curve), 2.6 mol % (green curve), and 5 mol % Cl-doped v-SiO_2_ (dark green curve). Schematics of Q^3^ and Q^4^ coordinated Si (in the center of the tetrahedron) are shown as insets in panel (b). Bridging oxygens (red) are shown to bridge other Si atoms. The bridging oxygen behind Si is shown as slightly transparent to aid in visualization of the tetrahedron. In the Q^3^ configuration, Si bonds to 3 bridging oxygen atoms and one Cl atom.

**Table 1:** Chlorine Content, Chemical Shifts, and Peak Intensities for the Samples and the Q^3^ and Q^4^ Peaks

chlorine content	Q^3^ peak chemical shift (ppm)	Q^4^ peak chemical shift (ppm)	calculated peak intensity ratio (Q^3^)	actual peak intensity ratio (Q^3^)
0 mol %	-	–111	-	-
2.6 mol % (±0.04)	–94.3	–111	2.7%	7%
5.6 mol % (±0.03)	–90.8	–111	6.0%	11%

Peak areas give relative proportions of the *Q* ^*x*^ species observed in NMR spectra. The percent abundance of *Q* ^*x*^ species is given by the following equation

2 $$\left[Q^{x}\;\left(\%\right)\right]=\frac{I_{Q^{x}}}{I_{Q^{3}}+I_{Q^{4}}}\times 100\%$$

where *I*_Q_^*x*^ is the peak area that corresponds to a given Q^*x*^ species, and *x* is the number of BOs. The integrated peak area of the −94 ppm peak in the 2.6 mol % Cl v-SiO_2_, taken with respect to the total integrated peak area, indicates that ∼7% of the Si atoms are Q^3^. As for the −91 ppm peak in the 5.6 mol % Cl v-SiO_2_, also corresponding to Q^3^, its area indicates that ∼11% of the total Si atoms are similarly affected by the Cl dopant. The Q^3^ peak area in the 2.6 mol % Cl sample is close to 2.5× the concentration of Cl in the sample. However, because the Q^3^ peak is less distinguishable in this spectrum, it is more difficult to extract precise peak amplitude, width, and chemical shift, leading to higher uncertainties in the estimated peak area and hence Q^3^ population with 2.6 mol % Cl. The Q^3^ peak in the 5.6 mol % Cl sample is more pronounced, and its fitted area is within error (signal-to-noise) of 2 times the concentration of Cl in that glass. Thus, these results consistently indicate that each Cl atom added to the silica network leads to a perturbation of two Si atoms (i.e., Q^3^ peak area scales with 2× the Cl concentration).

In halogen-doped silica glass, these Q^3^ species are widely considered to result from terminal bonds caused by the incorporation of the monovalent anion.^8^ Table 1 shows area ratios of the Q^3^ peak with the total peak area (amplitude of Q^3^ and Q^4^) and chemical shift at −94 ppm for 2.6 mol % and −91 ppm for 5.6 mol % Cl. These peak areas are higher than would be expected, as the relative amounts of components are 2.7% Cl/97.3% SiO_2_ and 5% Cl/95% SiO_2_ in the respective v-SiO_2_ samples. If we assume all Cl atoms are only singly bonded to Q^3^ silicon atoms, the computed peak area ratio is 2.7 for the 2.6 mol % Cl sample and 6.0 for the 5.6 mol % Cl sample. Why then are the measured Q^3^ abundances so high when the plausible explanation for the formation of a Q^3^ Si species is the bonding of a Cl atom to one Si with three B–O bonds? If instead each Cl is bonded to two Q^3^ silicon atoms, we would expect 5.3 and 11.9% Q^3^ ^29^Si in the 2.6 and 5.6 mol % Cl v-SiO_2_. The measured and calculated values are within the signal-to-noise error when the strength of the Q^3^ peak is taken into consideration. We propose that a single Cl atom, unlike the Si–F-terminating groups in the analogous F-doped silicas, bridges two Q^3^ Si atoms by replacing 2 BO; this configuration can explain the overabundance of Q^3^ species in the Cl-doped v-SiO_2_.

The existence of a bridging monovalent anion is highly unusual, and so the possibility of other Q^x^ species must be considered before concluding that the bridging Cl configuration explains these data. Of particular importance is the possibility that the second downshifted peak is Q^2^ speciation because the Q^3^ peak in F-doped v-SiO_2_ is located at −103 ppm.^3^ However, there are two lines of evidence that eliminate the possibility of a Q^2^ peak. First, for Q^2^ to appear in the spectra, we would need to see a peak that is even smaller than the shoulder feature in the 2.6 mol % sample, simply because a Q^2^ unit requires twice as much Cl as each Q^3^ unit. If all bridging Cl atoms formed Q^2^ species, its peak would be smaller than the error from signal noise. Second, assigning this peak to a Q^2^ species would require a much smaller, narrower peak, as the peak width corresponds to the distribution of BO bond angles (parameters in Supporting Information, Data S2–S4). The difference between the locations of Q^3^ in the F- and Cl-doped v-SiO_2_ samples is consistent with the interpretation that F has a terminal (and therefore shorter) bond and that Cl is hypervalent and forms longer bridging bonds between two silica tetrahedra.

The existence of bridging Cl atoms is also consistent with recent work on the structural silicate network properties in halogen-doped v-SiO_2_.^17^ Raman spectroscopy (Figure 2a) coupled with MD simulations (Figure 2b and c) show that average tetrahedral ring sizes in F- and Cl-doped v-SiO_2_ shift from larger, ≥5-member tetrahedral rings in undoped v-SiO_2_ to favor smaller, 3- and 4-member tetrahedral rings in the halogen-doped glasses.^17^ Changes in the network induced by addition of F or Cl result in fewer large rings and are compensated by increases in small (3- and 4-member) rings. Decreases in ≥ 5-member rings, which are correlated to decreases in intensity in the R-band (Si–O–Si symmetric bending at ∼440 cm^–1^), are more pronounced in the Cl-doped v-SiO_2_, indicating that at the same concentration Cl leads to higher connectivity relative to F.

**Figure 2:**
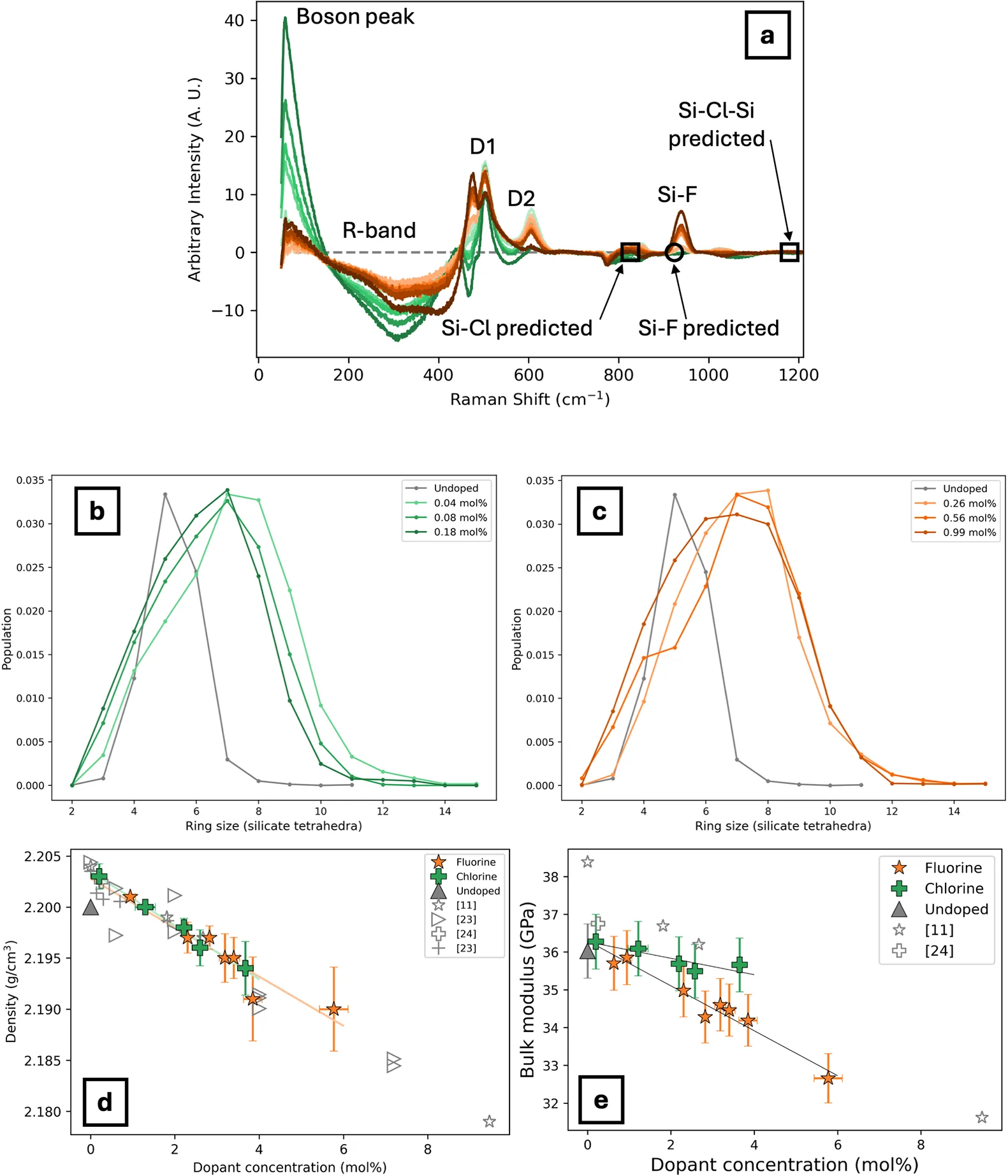
(a) Raman difference spectra of F- and Cl-doped v-SiO_2_, with predicted F peaks (circles) and Cl peaks (squares). (b) and (c) MD simulations of F- and Cl-doped v-SiO_2_. (d) Measurements of the density of F- and Cl-doped v-SiO_2_.^11,23,24^ (e) Resonant ultrasound spectroscopy measurements of the bulk modulus of F- and Cl-doped v-SiO_2_. Figure modified from ref 17. The calculated peak locations are highlighted on both panels (a) and (b) for comparison.

The plausibility of the bridging Cl is hampered by a single electronic vacancy in its valence shell. Consequently, halogens usually ionically or covalently bond with only one other atom; for example, the addition of F interrupts the silicate network by the formation of terminal bonds.^3^ The Si–F stretching vibration appears in Raman spectra at 945 cm^–1^ in v-SiO_2_ (Figure 2a).^25^ If Cl is bonded to Si in the same terminal configuration as F, it should have an analogous Raman band. Si–Cl stretching vibrations are predicted to appear at 826 cm^–1^ (Figure 2a). If there were a Si–Cl stretching vibration in our Raman spectra, the intensity of this feature should increase with Cl concentration as the Si–F peak does in the F-doped v-SiO_2_ spectra. In these locations, the intensities do not increase and, moreover, are approximately the same as those of the F-doped v-SiO_2_ at the same wavenumber, consistent with the interpretation that Cl does not form terminal bonds.

Bridging Cl vibrations are predicted at 1180 cm^–1^, which are not observed in our spectra and may indicate that the hypervalent Cl atom bonding to two Si atoms is Raman-inactive. The absence of a peak at 1180 cm^–1^ suggests that its signature could be obscured by more intense features, as bridging Cl might be indistinguishable from BOs in the medium-range order structural bands between 440 and 695 cm^–1^. The absence of a predicted peak at 826 cm^–1^ provides further evidence of the existence of bridging Cl because a hypervalent Cl atom would produce a net dipole moment configuration on the SiO_3_Cl unit and would thus be Raman-inactive. Furthermore, because F does not exhibit any evidence of bridging bonding, but Cl, a larger halogen with sp^3^ orbital hybridization, exhibits strong evidence for bridging Si atoms, it may follow that other large halogen elements, such as Br and I, may bridge Si atoms in v-SiO_2_.

Cross-linking in polymerized networks tends to create stronger structures, as in the case of vulcanized rubber.^26^ In a silicate network, we see that the formation of Cl–Si-bonded cross-links decreases the amount of larger, ≥5-member rings and is supported by decreasing intensity in the R-band in Raman spectroscopy and predicted in MD simulations (Figure 2a,b).^17^ Higher populations of small silicate rings correspond to decreased density^27^ and are consistent with observed decreases in density in our measurements (Figure 2D). Cl-doped v-SiO_2_ exhibits the same density decrease on a per-mole basis as F-doped v-SiO_2_ up through 3.7 mol %, despite Cl having nearly double the atomic mass of F.^17^ F and OH have similar mass and show similar decreases in density with concentration, which track with the replacement of one O atom per substitution.^17^ Cl, in contrast, substitutes for two O atoms. If Cl were nonbridging, it would likely have higher densities at comparable concentrations because of the heavier mass of Cl relative to F. Moreover, the observed differences in the elastic moduli further support bridging Cl. The moduli of Cl-doped v-SiO_2_ are consistently stiffer than the F-doped v-SiO_2_ despite having the same density (Figure 2d,e). In optical fiber applications where maintaining low fictive temperatures (and therefore low densities) is desirable, bridging Cl imparts more stiffness while retaining the same density behavior as F in v-SiO_2_.

The role of Cl in larger-scale structural disorder in v-SiO_2_ may be determined by analysis of the boson peak. Boson peak intensity is much higher in Cl-doped v-SiO_2_ in comparison to F-doped v-SiO_2_, but this analysis is largely qualitative. The boson peak is correlated to structural disorder and may also be related to the formation of percolation channels.^28^ Le Losq and co-workers studied alkali-doped aluminosilicate glass and found that higher concentrations of heavier elements result in larger boson peaks, and although a qualitative comparison, their findings are consistent with elevated boson peak intensity in Cl-doped v-SiO_2_ compared to that of F-doped v-SiO_2_.^23^ A nonclustered, bridging Cl configuration is shown in Figure 3a. Cl-“decorated” centers are composed of 4 terminal Cl–Si bonds arranged concentrically and are thought to be the primary defect—an SiO_4_ vacancy—stemming from Cl incorporation in unirradiated v-SiO_2_ (Figure 3b).^13,19^ Doubly coordinated Cl, bonded to one Si and coordinated to a Si atom with a free radical, has been identified in irradiated v-SiO_2_. These exist as triplet-like defects from decorated Cl centers (Figure 3c).^13^ Although the configuration in Figure 3c is only known to exist in irradiated v-SiO_2_, the coordination of Cl to two Si atoms—one covalently bonded, another coordinated by an electronic defect—is precedented. However, this coordination contains an electronic defect and noncovalent bonding, which has been ruled out in our study because this configuration would be Raman-active.

**Figure 3:**
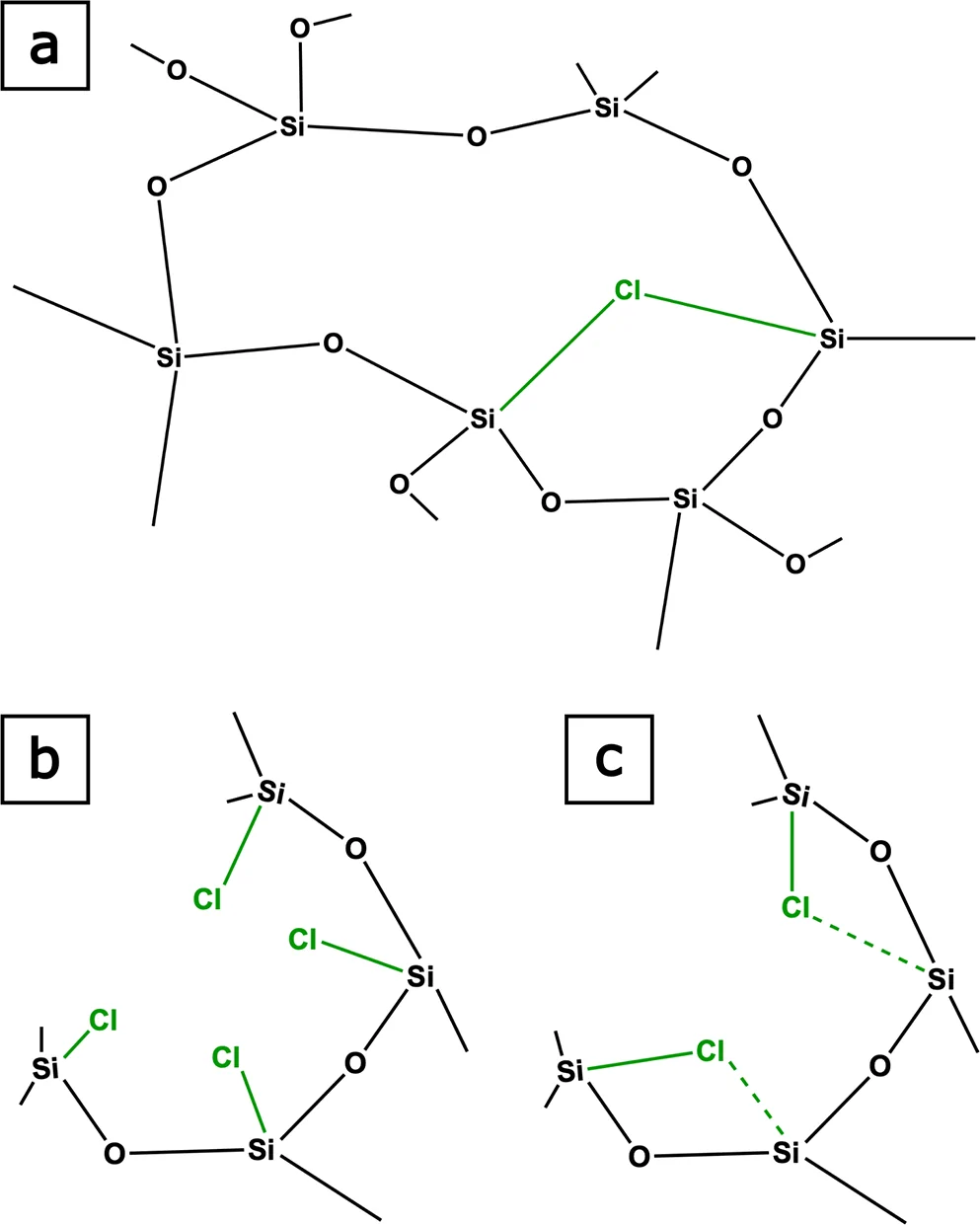
(a) A schematic depicting the covalent nature of the bridging Cl atom. The existence of the bridging Cl atom polymerizes, rather than depolymerizes, the network and closes off larger rings in favor of forming new, smaller rings. (b) A Cl-decorated center in unirradiated v-SiO_2_ and (c) a covalent bridging Cl center (modified after ref 13). The dots in panel (c) represent unpaired electrons.

A comparison of viscosity data between F- and Cl-doped v-SiO_2_ suggests that the doubly coordinated Cl configuration likely extends into the liquid phase. Viscosity varies most strongly with the concentration of Cl or F at lower temperatures. v-SiO_2_ with trace concentrations of Cl has the highest viscosities, and the highest concentrations of F or Cl exhibit the lowest viscosity values at a given temperature (Figure 4a,b, respectively). However, the extent to which the two halogens lower viscosity differs by 2 orders of magnitude (Figure 4c). The viscosity of the 1.1 mol % F v-SiO_2_ overlays the viscosity of a v-SiO_2_ sample containing 2.2 mol %, showing that a concentration of Cl equivalent to 2× F produces the same viscosity curve through a range of temperatures. We attribute this to the difference in bonding environments of the two halogens: the formation of terminal bonds in F-containing v-SiO_2_ and the formation of network-forming bridging Cl in Cl-containing v-SiO_2_. Terminal bonds reduce the viscosity more efficiently than bridging bonds because they reduce the connectivity of the silicate network, enabling faster atomic rearrangement in the liquid phase. Although viscosity measurements are not direct structural proof of a bridging Cl species, these data are consistent with the interpretations of spectroscopic and MD methods outlined previously. Furthermore, these viscosity results also support the persistence of bridging Cl in the liquid phase. Glass transition temperatures (*T*_g_) are shown in Figure 4a,b in shaded boxes, with extrapolation noted by dashed lines. As the archetypal strong liquid, v-SiO_2_ exhibits linear increases in log η with 1/*T*, and thus, *T*_g_ values are easily extracted by extrapolation of the linear fits to viscosity data. Since glass retains the structure of a kinetically hindered melt, chemical species and arrangements that exist in the glassy phase may also exist in the liquid phase. Early work on Raman spectra of v-SiO_2_ and its melts shows first-order similarities across *T*_g_, implying that the silicate network maintains much of its medium-range order structures.^29^ Since the existence of bridging Cl in the silicate network directly influences ring size populations, bridging Cl may then persist into the liquid phase. The existence of bridging Cl in the liquid phase implies that this configuration is stable at elevated temperatures above the glass transition temperature and into the liquid state.

**Figure 4:**
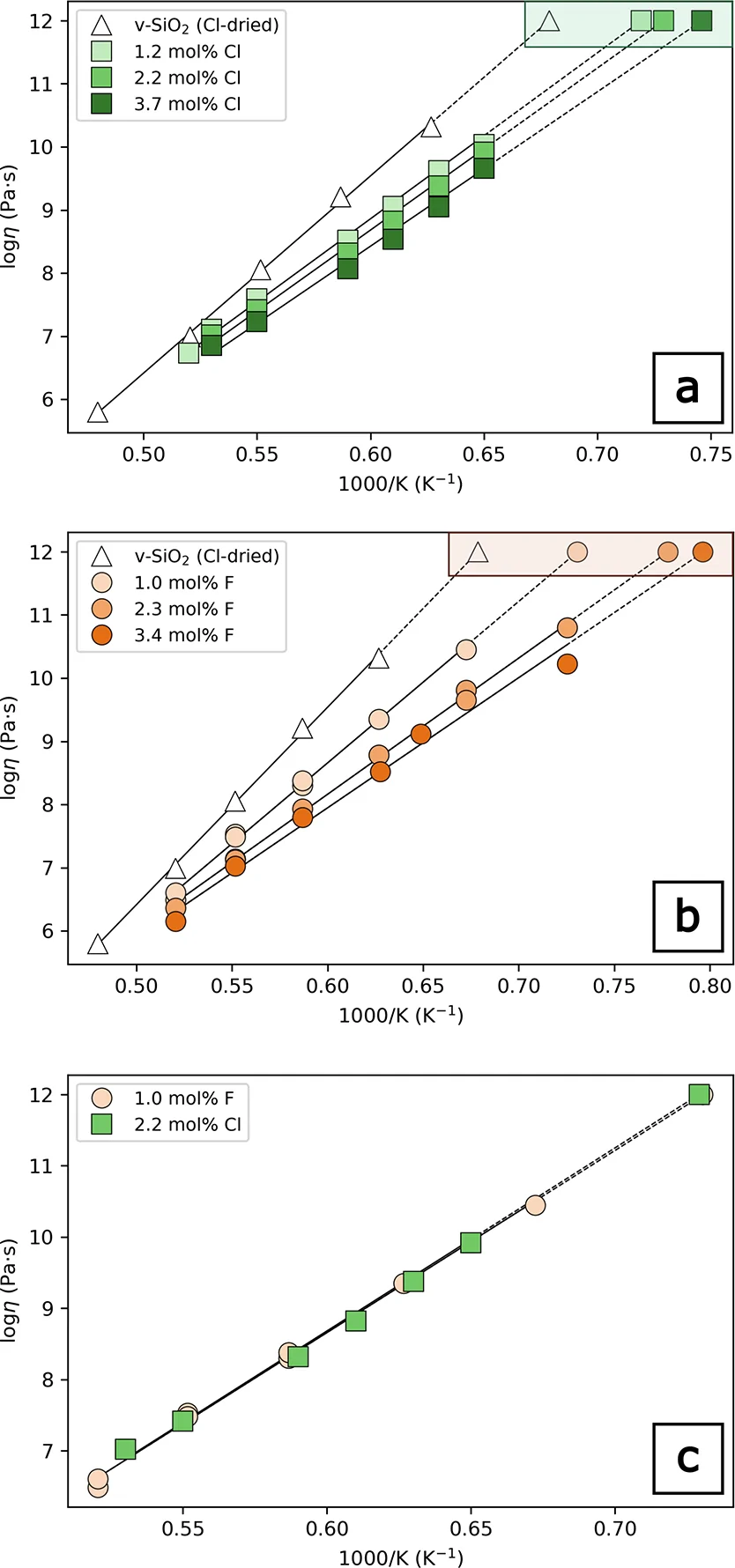
Results from one-atmosphere viscosity measurements on (a) Cl-containing and (b) F-containing v-SiO_2_. v-SiO_2_ contains trace Cl (full compositions in Supporting Information). Results for 1.0 mol % F and 2.2 mol % Cl are shown side-by-side in panel (c). *T*_g_ estimates are shown in panels (a) and (b) and are outlined by transparent boxes. They were calculated by linear extrapolation, as denoted by the dotted lines.

## Conclusions

Our results from ^29^Si MAS NMR and Raman spectroscopies are consistent with bridging Cl in v-SiO_2_. v-SiO_2_ samples containing 2.6 and 5 mol % Cl, respectively, exhibit peaks at −94 and −91 ppm, which we attribute to the formation of Q^3^ Si atoms. The integrated peak areas of the newly formed ^29^Si NMR resonances as a function of Cl content are twice as large as would be if each Cl atom were impacting the local environment of a single Si atom, as would be the case for terminal Si–Cl bonding that mimics that of F-doped silica. These integrated peak areas are consistent with a bridging Cl atom between two Q^3^ silicate tetrahedra. Evidence from elastic measurements and MD simulations is consistent with the formation of smaller rings in v-SiO_2_ that result from the effects of bridging Cl atoms in the silicate network, a structural view of Cl that is also supported by the lack of a terminal Si–Cl Raman peak. These findings have profound implications for fields such as materials science and geochemistry. For example, cross-linking via bridging Cl in v-SiO_2_ is a meaningful way to achieve stiffer silicate materials without changing other macroscopic properties such as density. Additionally, Cl is significantly more abundant on Mars than on Earth;^30^ bridging Cl may inhibit the compressibility and densification of Mars-derived silicate melts, perhaps influencing the evolution of its silicate crust and mantle.

## Supplementary Material





















## Notes

Electron microprobe analysis of samples for MAS NMR (XLSX)
MAS NMR spectra (TXT)
MAS NMR spectra (TXT)
MAS NMR spectra (TXT)
Viscosity data (XLSX)
Viscosity data (XLSX)
Viscosity data (XLSX)
Electron microprobe analysis of samples for viscosity (XLSX)
Electron microprobe analysis of samples for viscosity (XLSX)
Materials and methods, molecular dynamics simulations, Table S1, viscosity measurements, and Figure S1 (PDF)
