# Supplementary material for: Observation of Bridging Chlorine in Vitreous SiO2
Source: J Phys Chem B. 2026 Sep 29;130(41):10984–90. doi: 10.1021/acs.jpcb.6c02141 (PMC13679672; doi:10.1021/acs.jpcb.6c02141)
Supplement: Supplementary file 1 [file jp6c02141_si_010.pdf]

Supporting information

Observation of bridging chlorine in vitreous SiO_2

Lindsay M. Harrison[a*], Adam R. Sarafian[b], Randall E. Youngman[b], Craig D. Nie[b], Matthew E. McKenzie[b], and Alisha N. Clark[a*]

AUTHOR ADDRESS.

[a]Department of Earth Science, University of Colorado, Boulder, Boulder, CO 80309, USA

[b]Corning Incorporated, Science & Technology, Corning, NY 14831, USA

[*]Corresponding authors. Email: lindsay.harrison@colorado.edu; alisha.clark@colorado.edu

Table of Contents

Materials and Methods S3-S5
Molecular dynamics simulations S3
Table S1 .... S4
Viscosity measurements S4
Figure S1 .... S5

MATERIALS & METHODS

Molecular dynamics simulations.

Table S1: Element pair and LJ parameters.

Element Pair	Lennard Jones ε (eV)	Lennard Jones σ (Å)
**$Si^{+2.4}$ – $Cl^{-0.6}$**	3.949	2.08
**$Si^{+2.4}$ – $F^{-0.6}$**	5.973	1.7
**$O^{-1.2}$ – $H^{+0.6}$**	4.790	0.96

Viscosity measurements.

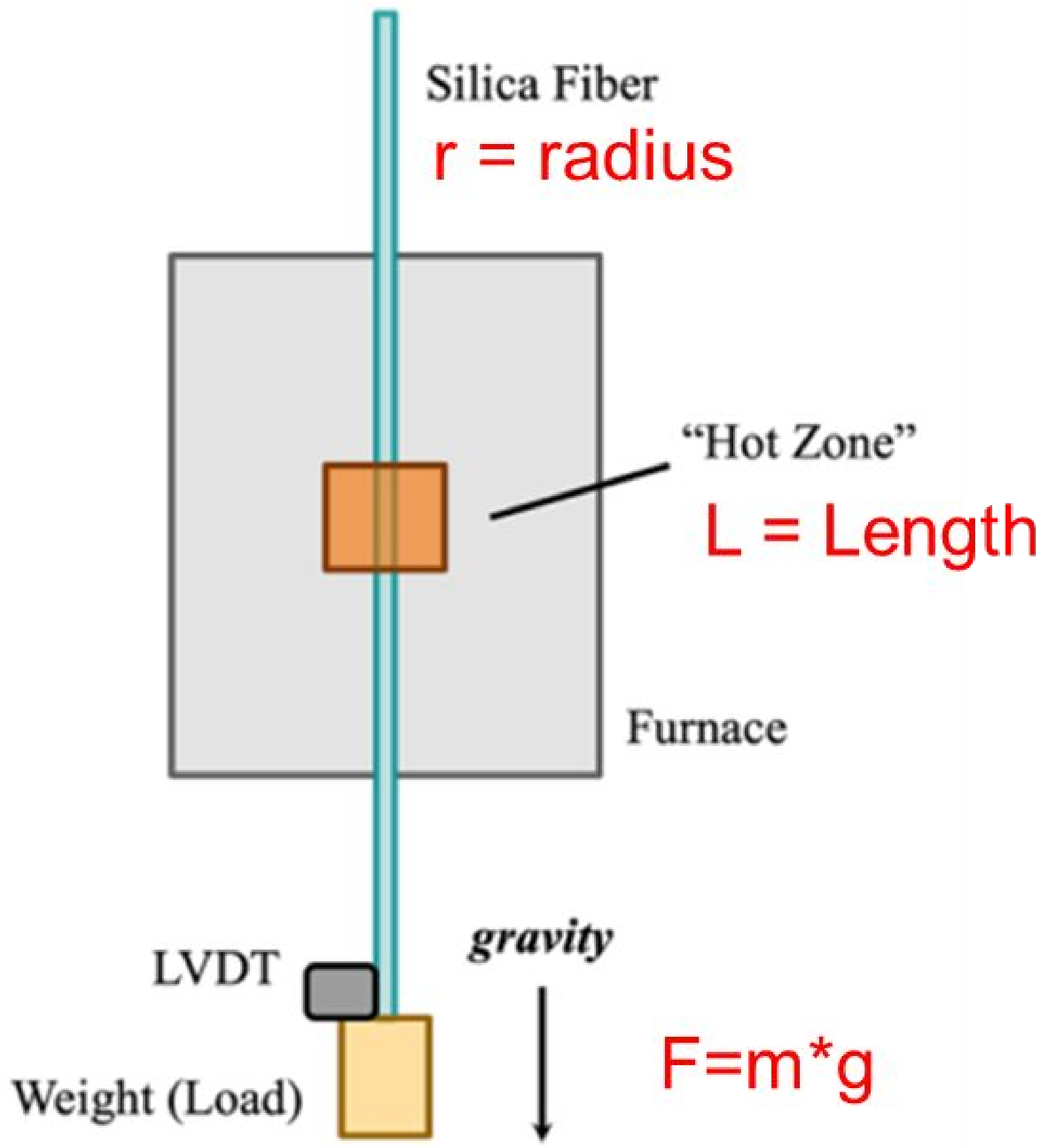

Figure S1. A schematic depicting the experimental apparatus to measure cane displacement. The silica fiber, or cane, is shown in teal.

Table S2: Glass transition temperatures and fitting parameters from log η vs 1000/T (1/K) plot.

**Dopant**	**Mol %**	**T_g (K)** at 10^{12} Pa s	**1000/T (1/K)**	**E_a (kJ/mol)**	**Fitting parameters** m	b (log η, Pa s)
SiO_2	0	1473	0.679	598.5	31.2	-18.2
Cl	1.2	1390	0.719	503.7	26.3	-15.9
Cl	2.2	1371	0.729	491.6	25.7	-15.7
Cl	3.7	1340	0.746	468.4	24.5	-15.3
F	1.0	1369	0.730	489.9	25.6	-15.7
F	2.3	1286	0.778	413.0	21.6	-13.8
F	3.4	1256	0.796	395.1	20.6	-13.4
